# Peak-Load-Regulation Nuclear Power Unit Fault Diagnosis Using Thermal Sensors Combined with Improved ICA-RF Algorithm

**DOI:** 10.3390/s21216955

**Published:** 2021-10-20

**Authors:** Yifan Wu, Kaiyu Wu, Wei Li, Jianhong Chen, Zitao Yu

**Affiliations:** College of Energy Engineering, Zhejiang University, Hangzhou 310027, China; 11727037@zju.edu.cn (Y.W.); marilyn@zju.edu.cn (K.W.); power@zju.edu.cn (J.C.); yuzitao@zju.edu.cn (Z.Y.)

**Keywords:** nuclear power unit, thermal sensor fault diagnosis, peak load regulation, blind source separation, random forest

## Abstract

Owing to the fact that large-scale peak-load-regulation nuclear power turbine units’ thermal signal is greatly influenced by background noise and has non-stationary and nonlinear characteristics, this paper proposes a new fault diagnosis method for thermal sensors based on an improved independent component analysis (Improved-ICA) algorithm and random forest (RF) algorithm. This method is based on independent component analysis (ICA), which is not capable of extracting components independently. Therefore, we propose the use of the maximum approximate information negative entropy optimization model in order to improve the ICA algorithm’s independent principal component extraction ability and obtain better non-Gaussian physical source signal separation results. The improved ICA algorithm is used for the blind source separation of the thermal parameters of peak-load-regulation nuclear power units. A series of stationary physical source functions and a series of non-stationary noise signals are obtained. Then, according to the specific signal format and data volume of the nuclear power parameter signal, the network parameters of the random forest algorithm are determined, giving rise to the fault diagnosis model. Finally, the real-time operation data of an 1121 MW nuclear power unit are used to complete the training and fault diagnosis of the random forest network and analyze the diagnosis results. The results indicate that the model can effectively mine the abnormal sample points of thermal parameters and classify the fault type of the thermal sensor during peak load operation of the nuclear power unit. The accuracy rate is found to be at the threshold of 99%.

## 1. Introduction

China, in recent years, has witnessed the growth of the number of nuclear power units put into operation and their share of power generation. In nuclear power units that participate in peak load regulation, their operating parameters deviate from the design values, and the stress, thermal expansion, and thermal deformation of each component of the unit will change. Thermal system sensors work in harsh environments and thus, are more prone to failure. The thermal signal of large-scale peak shaving nuclear power turbine units will experience considerable disturbance due to background noise, as well as non-stationary and non-linear. The traditional data mining technology finds it difficult to analyze this kind of data effectively, which impedes the extraction, analysis and diagnosis of fault information. Hence, research on the signal separation technology and feature extraction technology used for the thermal signals of nuclear power units, and obtaining accurate abnormal signals from the monitoring signals, are the basis of sensor fault diagnosis in thermal systems. A recent serious accident was caused by the failure of a thermal sensor. The thermal sensor had a serious precision degradation fault, but the signals were misjudged as peak-load-regulation. Due to wrong judgment and operation, the thermal parameters exceeded the threshold, causing serious damage to the power units and bringing huge economic losses. Affected by the above problems, the operation safety of peak-load-regulation power plants is facing great challenges, especially peak-load-regulation nuclear power stations.

Chinese peak-load-regulation nuclear power unit is an innovative engineering problem that has emerged in recent years. Most research on the peak load operation of nuclear power units mainly focuses on the aspects of economy and feasibility. Shi et al. [1] conducted an in-depth study on the parameter changes of nuclear power units during peak load regulation. It was found that a change in any of the parameters (power, flow, pressure, temperature, etc.) during peak load operation will directly affect the operation status of the main steam turbine, as well as the sensors in the thermal system. Consequently, abnormal data fluctuations of the sensor largely originate from two sources: abnormal thermal system parameters caused by the load fluctuation of peak load regulation and the abnormal parameters of the thermal system caused by sensor faults. Zhao et al. [2] pointed out that it is now difficult to ensure the baseload operation of nuclear power units previously designed and operated in accordance with the basic load mode. Therefore, it is necessary to consider adjusting the characteristics of the equipment to cope with the peak-regulation pressure, to enable it to gradually participate in the peak load operation. Song et al. [3] completed a study on the feasibility of nuclear power peak regulation. Some nuclear power units in China have participated in short-term power reduction peak regulation operations during special festivals, such as national day and Spring Festival, and the results of the peak regulation operation are sound. Peng et al. [4] and Gao et al. [5] who studied nuclear power abroad share a common view that the power fluctuations in European countries are too small to reach the level where nuclear power units are required to participate in peak load operation. Although nuclear power can regulate peak loads, it still maintains the baseload operation for the purposes of economy and safety. Wu et al. [6], who studied the peak-load-regulation operation and vibration fault of large-scale thermal power plants, proposed a vibration fault diagnosis model based on the PCA-HKNN (Principal Component Analysis-Hierarchy K Nearest Neighbor) algorithm and carried out experiments with the data of 1000 MW thermal power units. The experimental outcomes illustrate that the model can efficiently diagnose the peak shaving fault of thermal power plants. Li et al. [7] improved auto-associative kernel regression (AAKR) with the sequential probability ratio test (SPRT). Simulations of fault detection and identification in the sensors and components of the reactor coolant system of the Qinshan nuclear power plant were carried out, with the results demonstrating the effectiveness of the proposed model.

International scholars attach great importance to the nuclear industry and nuclear power market. Zhang et al. [8] studied the nuclear power market in North America and proposed a rapid method for the calculation of nuclear power performance, which can also analyze the evaluation index of the nuclear power market. Shorthill et al. [9] put forth the Bayesian and HRA-Aided Method for the Reliability Analysis of Software (Bahamas) for analyzing nuclear power in North America. Based on human reliability analysis (HRA), and common cause failure (CCF), the algorithm can diagnose the vibration failure and human factor faults of nuclear power. Reichenberg et al. [10] studied the performance of European nuclear power plants and proposed the improved conditional value at risk (CVaR) algorithm to evaluate the transient adjustment capability of European nuclear power plants. Choi et al. [11] analyzed the future development mode of nuclear power in South Korea. The future of nuclear power in South Korea is based on the safety of the nuclear industry and the capacity of nuclear energy absorption. Tripathi et al. [12], meanwhile, described the design-time methodology that can be used to map and analyze the system security qualitatively and quantitatively using Stochastic Petri nets and their fundamental properties. The effectiveness of this method was verified by the study of an Indian nuclear power plant.

Recent studies in this field only briefly discuss the feasibility and necessity of the participation of nuclear power units in peak load regulation and lack any in-depth analysis of the thermal sensor fault diagnosis of peak-load-regulation nuclear power units. Thermal sensors are very important instruments in nuclear power plants; their accuracy and effectiveness are closely related to the efficiency and safety of nuclear power units.

In terms of sensor fault diagnosis, Li et al. [13] carried out the research on the thermal sensor fault diagnosis of thermal power units and used empirical mode decomposition (EMD) to decompose the sensor signal to obtain the eigenvalue of the signal. A fault diagnosis method for sensor signals based on deep learning was proposed by Xing et al. [14]. This method employs the LSTM algorithm to optimize the CNN model and improve its ability to diagnose the mixed faults of sensors. Yu et al. [15,16] improved the EMD by using probabilistic neural networks on the basis of Li et al. [13]. This method focuses on the fault diagnosis of temperature sensors in thermal power units. Yu et al. [17] proposed a double improved principal component analysis model to improve the safety of sensors in nuclear power plants. The Corrected Reconstruction Algorithm (CRA) was used to facilitate the accuracy of the reconstruction. The second improvement is in the form of a cyclic monitoring PCA (CPCA) model that can detect multi-sensor failures. A modification of the traditional AAKR method that enhances the signal reconstruction robustness was proposed by Baraldi et al. [18]. The obtained results reflect an improvement in the early detection of abnormal conditions and the correct identification of the signals responsible for triggering the detection.

In other industrial fields, Sun et al. [19] proposed a blast furnace fault diagnosis model based on an independent component analysis algorithm, this model has a high accuracy of non-Gaussian fault diagnosis of a steel-making blast furnace. Wan et al. [20] implemented a multi-block independent component analysis algorithm for correcting the random gross error of chemical sensors. Peng et al. [21] established a vibration fault diagnosis model of high-speed machinery based on machine vision and ICA blind source separation. Sun et al. [22] proposed an adaptive selection method for nonlinear function in ICA. This method tackles the separation of rolling bearing fault signals under multiple vibration sources. In some fields, information entropy is widely used in data validation, outlier analysis, weight determination and so on. Dang et al. [23] proposed a variable mode decomposition method based on information entropy. This method could extract the characteristic signal from the vibration signal of the wind turbine gearbox and give the information weight value, so as to improve the calculation speed of the neural network. Wang et al. [24] proposed a sensor fault diagnosis method of a hydraulic condition monitoring system based on information entropy and the K-2 algorithm. Information entropy and the K-2 algorithm were used to improve the search strategy, so as to optimize the generation method of Bayesian network structure and improve the calculation accuracy and speed.

In this paper, we studied the thermal sensor fault of a large-scale peak-load-regulation nuclear power turbine generator set and propose a new thermal sensor fault diagnosis method based on an improved independent component analysis algorithm and the random forest algorithm. Signal analysis technology, data mining technology, feature recognition technology and machine learning technology are used in the manuscript. Two main models are studied and analyzed, including the blind source separation (BSS) model and the tree classification model. The ICA algorithm is used to realize the BSS model, and the RF algorithm is selected for the tree classification model. Taking RF as an example, when the noise of data is too large, RF is prone to overfitting and the accuracy is not high. Therefore, ICA is used for signal analysis and data mining to reduce the noise signal in the original signal and enhance the RF. The mathematical calculation of the cost function of traditional ICA is difficult, and the signal separation speed is low. The maximum approximate information negative entropy method can reduce the calculation difficulty and improve the calculation speed. This method is used to strengthen ICA in this manuscript, and its rationality is proved.

The research object and data source is a 1121 MW peak-load-regulation nuclear power unit. Firstly, the collected data samples are analyzed and an ICA blind source separation model is established. Then, the maximum approximate information negative entropy algorithm is used to optimize the non-Gaussian judgment function in the ICA algorithm with the purpose of improving the independent principal component extraction ability of the ICA. The improved-ICA algorithm is employed to complete the blind source separation and feature extraction of the original signals. Finally, the RF algorithm is used to complete the thermal sensor fault diagnosis of the peak-load-regulation nuclear power unit, and the diagnosis results are evaluated based on the actual operation experience and feedback data of the nuclear power unit.

## 2. Analyzing Techniques

### 2.1. Blind Source Separation

#### 2.1.1. Independent Component Analysis Algorithm

ICA, a blind source separation signal analysis and data processing technology can separate the source signal from the observed mixed signal by an inverse transformation without the characteristics and mixed conditions of the source signal. Supposing that in the presence of noise $NS_{i}\left(t\right)$, n observation signals $x_{i}\left(t\right)$ are composed of m physical signal sources $s_{ij}\left(t\right)$ in some way, and the m physical signal sources are statistically independent, the n observation signals can be expressed as follows:

(1) $$x_{i}\left(t\right)=NS_{i}\left(t\right)+\sum_{j=1}^{m}a_{ij}s_{j}\left(t\right)$$

Where, $x_{i}\left(t\right)$ is the observation signal, $a_{ij}$ is a linear mixed function, $s_{ij}\left(t\right)$ is the physical source signal, and $NS_{i}\left(t\right)$ is the noise signal. For a certain $x_{a}\left(t\right)$, Equation (1) can be written as a matrix pattern:

(2) X=AS+M

(3) $$S=A^{-1}X-A^{-1}M$$

Where X is the observation signal matrix, M is the noise matrix, A is the mixed matrix, and S is the independent component matrix.

In engineering practice, in order to simplify the calculation, noise signal M is usually regarded as a special physical source signal. Suppose that the observed signal X obtained by the sensor is a combination of the physical source signal S including the noise signal, and the physical source signal cannot be obtained. Let $A^{-1}=B^{T}$, then the above formula can be rewritten as:

(4) $$X=A^{-1}S=B^{T}S=\underset{i}{\sum }b_{i}x_{i}$$

ICA, based on the principle of statistical independence, is usually implemented using a non-Gaussian maximization method which shows that the results of a group of random variables whose mean and variance are of the same order of magnitude must be close to a Gaussian distribution. Therefore, the problem boils down to finding a set of transformation parameters $b_{i}$ to maximize the non-Gaussian property of X.

If the observed signal is a linear combination of multiple independent physical source signals, then the observed signal is closer to a Gaussian distribution than the physical source signal. When the non-Gaussian property of the separated physical source signal reaches its peak, the separation can be considered to be optimal. Improving the independent component extraction algorithm is a sound foundation by which to improve the separation effect of the ICA algorithm.

#### 2.1.2. Improved-ICA Algorithm

Although the ICA algorithm can be widely used to extract non-Gaussian independent components, due to the use of orthogonalization, the ICA algorithm has the following drawbacks:

(1) The selection of the independent principal component has a direct impact on the effectiveness of the ICA algorithm;

(2) In the ICA algorithm, the different ranking weights of the same batch of independent principal components have a certain impact on the effectiveness of the ICA algorithm.

When the ICA algorithm is used to monitor the working state of the process, the number of selected independent principal components and the order of each independent principal component will affect the final result, thus affecting the accuracy of the fault detection. In particular, the peak load regulation of a nuclear power unit will lead to large changes in several thermal parameters, creating a burden on the determination of independent principal components, and the separation effect will experience an obvious dip.

Therefore, it is necessary to optimize ICA, and finding a new model to optimize the selection and ranking of independent elements is an important research component of optimizing ICA. The selection and ranking of independent components is actually the method of component analysis. At present, the principal component analysis, the factor clustering and the Gaussian sigma criterion clustering are commonly used. This paper combines the information entropy analysis technology of signal theory to improve the ICA algorithm.

Information entropy is a basic concept in information theory. The entropy of random variable can be interpreted as the amount of information contained in the observed variable. The more random and unpredictable a variable is, the greater its entropy is. Strictly speaking, entropy is related to the coding length of random variables. In fact, under some simple assumptions, entropy is the coding length of random variables. The information entropy H of random variable Y is defined as follows:

(5) $$H\left(Y\right)=-\underset{i}{\sum }P\left(Y=a_{i}\right)logP\left(Y=a_{i}\right)$$

In information theory, there is a basic conclusion: among all random variables with equal variance, the Gaussian variable has the largest entropy which means that the information entropy can be used as a measure of non-Gaussianity. In fact, it shows that the Gaussian distribution is the most random distribution of all distributions. The more concentrated the distribution of random variables is, the smaller the entropy is. In order to obtain a non-Gaussian measure with zero and permanent nonnegative Gaussian variables, the negative entropy is usually used. The definition of the negative entropy J is as follows:

(6) $$J\left(y\right)=H\left(y_{Guass}\right)-H\left(y\right)$$

where J is the negative entropy of information, $y_{Guass}$ is a Gaussian random variable with the same variance as y, and H is the information entropy.

According to statistical theory, the negative entropy is the best estimation of whether something is non-Gaussian. However, the calculation of the negative entropy is very complex and requires the estimation of the probability density function through defining the negative entropy. The negative entropy algorithm is still a theoretical algorithm that needs to be optimized.

Based on the principle of the maximum information entropy, the improved-ICA algorithm is proposed in this paper. The maximum entropy principle assumes that the information does not make any unknown assumption and treats the unknown event as an equal probability event. The definition of the maximum approximate negative entropy is as follows:

(7) $$J_{max}\left(y\right)=\overset{p}{\underset{i=1}{\sum }}k_{i}{\left[E\left\{G_{i}\left(y\right)\right\}-E\left\{G_{i}\left(v\right)\right\}\right]}^{2}$$

Where $k_{i}$ is a positive constant, y and v are Gaussian variables with a mean value of 0 and a variance of 1, and $G_{i}$ is a non-quadratic function.

In the ICA algorithm, an important part is the non-Gaussian judgment method of a signal. The negative entropy has the ability to distinguish the non-Gaussianity in the statistical sense, but it needs to be strictly mathematically proved before taking the negative entropy as the cost function of ICA.

If the negative entropy can become the non-Gaussian criterion in the ICA algorithm, the entropy maximum theorem must be satisfied: among all distributions with the same covariance matrix, the entropy of Gaussian distribution is the largest. It can be proven with the Kullback–Leibler divergence equation.

Assuming that $p_{g}\left(x\right)$ is a joint probability density function of Gaussian distribution, $p\left(x\right)$ is a joint probability density function distributed in any way. $p\left(x\right)$ and $p\left(x\right)$ have the same covariance matrix. The Kullback–Leibler divergence between them is shown in Equations (8) and (9):

(8) $$D\left(p\left(X\right)∥p_{g}\left(x\right)\right)=\int p\left(X\right)\ln\frac{p\left(X\right)}{p_{g}\left(x\right)}dx$$

(9) $$\int p\left(X\right)\ln p_{g}\left(x\right)dx=\int p_{g}\left(x\right)\ln p_{g}\left(x\right)dx$$

The logarithmic function satisfies the following relationship, as shown in Equation (10):

(10) $$\int p\left(X\right)\ln\frac{p\left(X\right)}{p_{g}\left(x\right)}dx=-\int p\left(X\right)\ln p\left(x\right)dx+\int p\left(X\right)\ln p\left(X\right)dx$$

The definition of information entropy has been explained in Equation (5), and Equation (11) can be obtained:

(11) $$H=-\overset{+\infty }{\underset{-\infty }{\int }}p\left(X\right)\ln p_{g}\left(x\right)dx$$

Equation (12) can be obtained from Equation (8) to Equation (11):

(12) $$D\left(p\left(X\right)∥p_{g}\left(x\right)\right)=H_{G}\left(X\right)-H\left(X\right)$$

The Kullback–Leibler divergence equation is nonnegative. Then Equation (12) meets the following conditions, as shown in Equation (13):

(13) $$H_{G}\left(X\right)-H\left(X\right)\geq 0\Rightarrow H_{G}\left(X\right)\geq H\left(X\right)$$

Equation (13) proves the maximum entropy theorem, that is, in all distributions with the same covariance matrix, the entropy of the Gaussian distribution is the largest. In other words, under certain conditions, a specific distribution can be found, which has the maximum information entropy, and this distribution is just the Gaussian distribution. The definition of negative entropy is shown in Equation (14):

(14) $$J\left(X\right)=H_{G}\left(X\right)-H\left(X\right)=D\left(p\left(X\right)∥p_{g}\left(x\right)\right)$$

In Equation (14), if and only if $p\left(X\right)$ satisfies Gaussian distribution, $J\left(X\right)=0$. An important property of the negative entropy is that it remains unchanged for reversible linear transformation. The property of the information entropy is that it remains unchanged for orthogonal transformation. Compared with the entropy theory, the condition of the negative entropy is more relaxed. In ICA, this property makes it possible to take the maximum approximate negative entropy as the cost function, so the effectiveness of the maximum approximate negative entropy criterion in the ICA algorithm is valid.

The cost formula of the original entropy method is shown in Equation (6), but mathematically, Equation (6) is difficult to calculate. In general, some empirical formulas will be used to simplify Equation (6), as shown in Equation (15):

(15) $$J\left(y\right)\approx \frac{1}{12}E{\left\{y^{3}\right\}}^{2}+\frac{1}{48}kurt{\left(y\right)}^{2}$$

The error of the empirical formula is often large and the empirical formula is not a good cost function. Therefore, in the maximum information negative entropy, the Newton method is introduced to obtain the maximum approximate solution.

Assuming $y=B^{T}x$, the information negative entropy formula satisfies the following constraints, as shown in Equation (16):

(16) $$\frac{\delta J_{G}\left(B\right)-\beta \left({||B||}^{2}-1\right)}{\delta B}=2E\left\{xG^{\prime }\left(B^{T}x\right)\right\}-2\beta B=0$$

Suppose $B_{0}$ is the optimal solution, then the following equation is satisfied, as shown in Equation (17):

(17) $$\beta =E\left\{B_{0}^{T}xG^{\prime }\left(B^{T}x\right)\right\}$$

The problem is simplified to solving the root of the equation. According to Newton’s method, if the tangent of the curve is used to approximately describe the curve, the root of the tangent equation is the root of the curve equation. If not, continue to perform the Newton method until convergence is reached. The formula of Newton method is shown in Equation (18):

(18) $$x_{n+1}=x_{n}-\frac{f\left(x_{n}\right)}{f^{\prime }\left(x_{n}\right)}$$

After the parameters are substituted, Equation (18) is transformed into Equation (19):

(19) $$B_{n+1}=B_{n}-\frac{E\left\{xG^{\prime }\left(B_{n}^{T}x\right)\right\}-\beta B_{n}}{E\left\{xG^{\prime \prime }\left(B_{n}^{T}x\right)\right\}-\beta E\left\{xx^{T}\right\}}$$

Continue the Newton method to get the recurrence equation. $B_{n+1}$ is replaced by $B^{*}$, and $B_{n}$ is replaced by B. It is shown in Equation (20):

(20) $$B^{*}=B-\frac{E\left\{XG_{i}\left(B^{T}X\right)\right\}-\beta B}{E\left\{G_{i}\left(B^{T}X\right)\right\}-\beta }$$

(21) $$B=\frac{B^{*}}{||B^{*}||}$$

Since the B value is updated, $\beta =E\left\{B^{T}XG_{i}\left(B^{T}X\right)\right\}$. The iterative formula for this is:

(22) $$B^{*}=E\left\{XG_{i}\left(B^{T}X\right)\right\}-E\left\{G_{i}\left(B^{T}X\right)\right\}B$$

**Figure 1:**
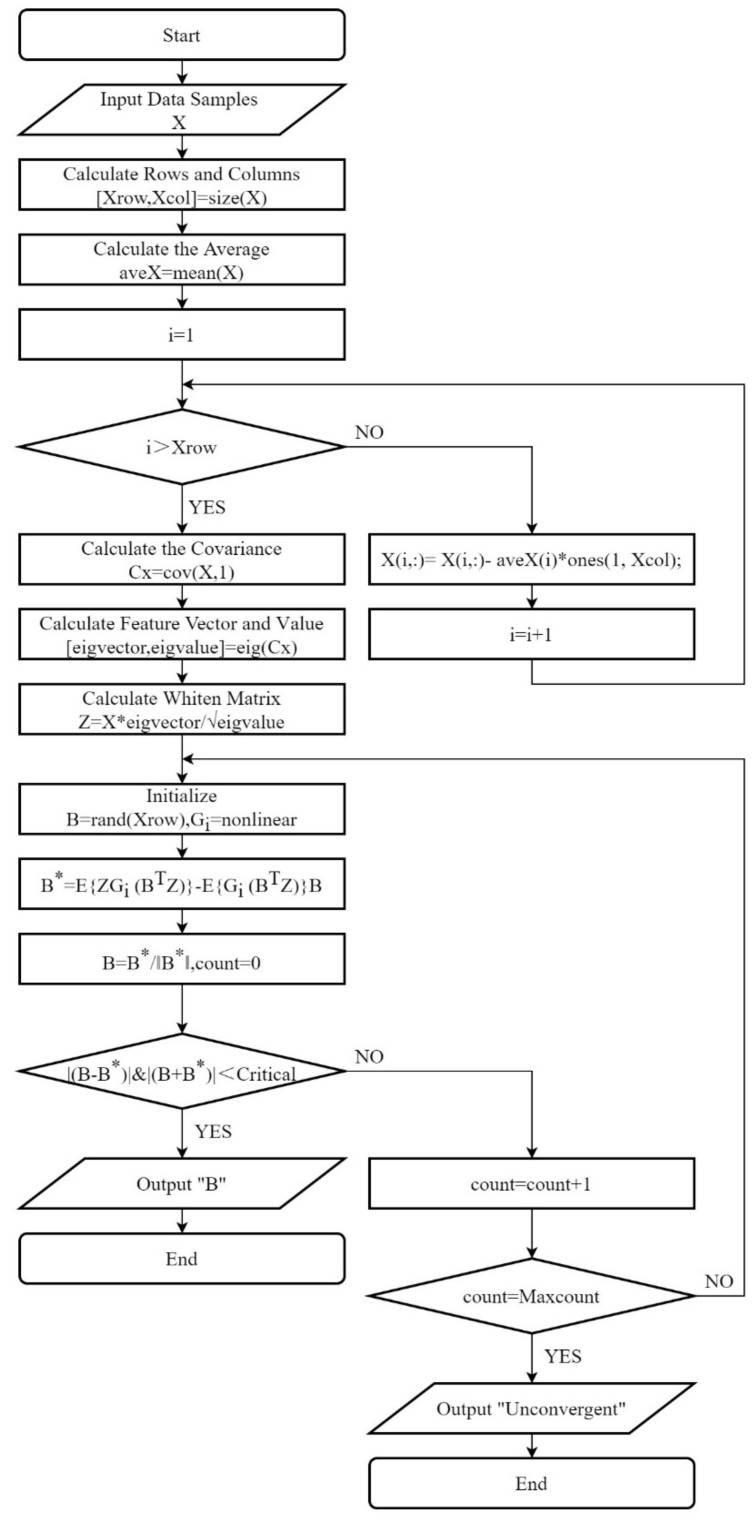
Improved ICA Flow Chart.

The de-averaging process should be performed before the calculation. De-averaging means that each dimension subtracts the mean value of the corresponding dimension so that each dimension of the input data is centered to 0. After de-averaging, the data distribution is near the coordinate axis. The network parameters are generally initialized randomly before network training. If the data has been de-averaged, the function obtained by random initialization can approach the objective function faster. In addition, if the data has not been de-averaged, the algorithm is easy to fall into local optimization and overfitting can easily occur. The de-averaging code is described in the Appendix A, Pseudo Code, Step 1 to Step 4. The algorithm steps are as follows and the flow chart is shown in Figure 1:

(1) Whiten X to obtain whiten matrix Z

(2) Initialize B

(3) Set $B^{*}=E\left\{ZG_{i}\left(B^{T}Z\right)\right\}-E\left\{G_{i}\left(B^{T}Z\right)\right\}B$ and select the nonlinear function $G_{i}$

(4) Set $B=\frac{B^{*}}{||B^{*}||}$. If it does not converge, return to step 3

(5) The results of the observed signal are obtained as physical source signal $S=\left[s_{1},\;s_{2},s_{3},\ldots ,\;s_{m}\right]$ and noise signal M.

### 2.2. Random Forest Algorithm

The random forest is an integrated method that combines multiple decision trees. The random forest algorithm uses the bootstrap sampling method to extract multiple samples from the original samples and establishes the decision tree model according to each bootstrap sample. Then, the predictions of multiple decision trees are integrated, and the final results are obtained by voting. Random forest regression can be regarded as a strong predictor integrating many weak predictors. Since the three common construction methods for the random forest, including ID3, CART, and C4.5, are top-down greedy algorithms [25], one of the most important bases for establishing a random forest model is the minimum mean square deviation.

Minimum mean square deviation (MMSE) is the basis of selecting features and dividing points. For any partition feature A, the data set divided on both sides of any partition point a is called $D_{1}$ and $D_{2}$. The corresponding feature and eigenvalue partition points corresponding to the minimum mean square deviation of each set of $D_{1}$ and $D_{2}$ and the minimum sum of the mean square deviations of $D_{1}$ and $D_{2}$ are obtained. The expression for this is:

(23) $$\underset{A,a}{min}\left[\underset{c_{1}}{min}\underset{x_{i}\in D_{1}\left(A,a\right)}{\sum }{\left(y_{i}-c_{1}\right)}^{2}+\underset{c_{2}}{min}\underset{x_{i}\in D_{2}\left(A,a\right)}{\sum }{\left(y_{i}-c_{2}\right)}^{2}\right]$$

$c_{1}$ is the sample output mean of $D_{1}$ data set and $c_{2}$ is the sample output mean value of $D_{2}$ data set.

**Figure 2:**
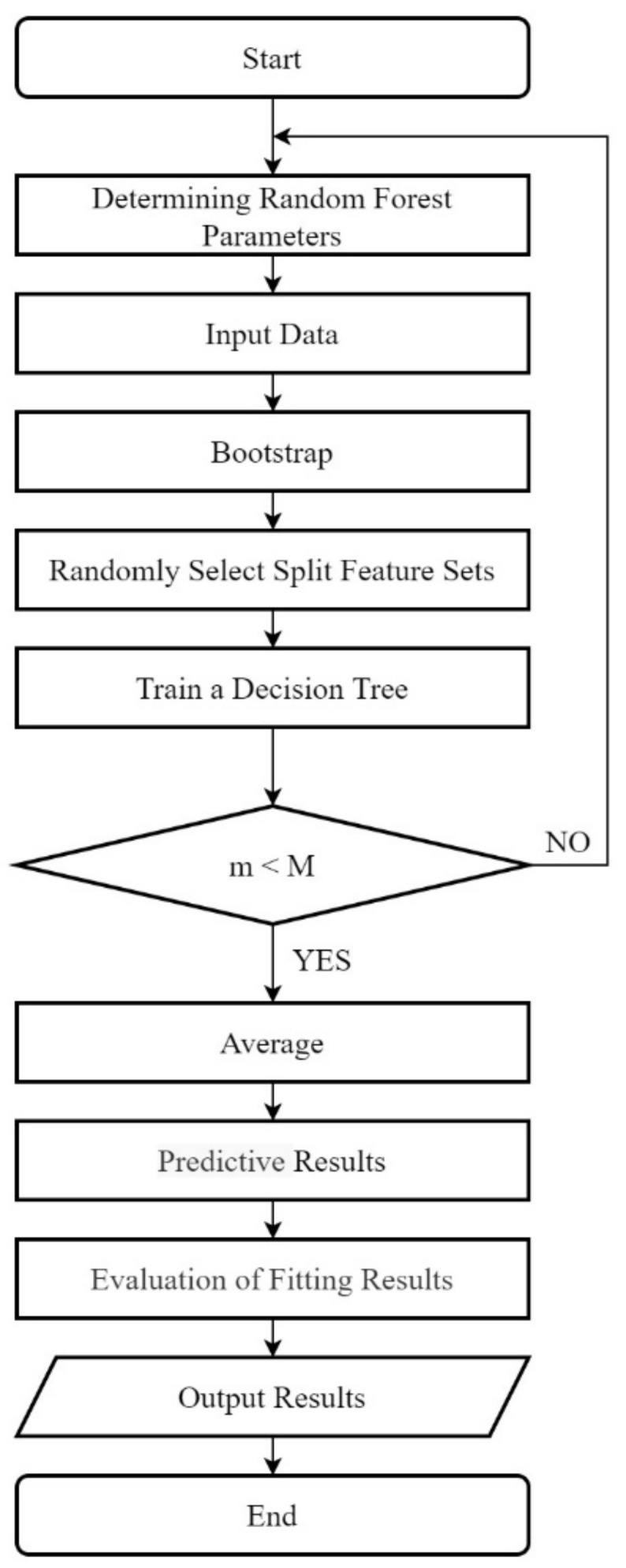
Random Forest Flow Chart.

The steps taken to establish the random forest regression model are as follows, and the flow chart is shown in Figure 2.

(1) N is defined as the number of training samples and M is defined as the number of features.

(2) The input feature number m is used to determine the decision result of a node on the decision tree. It should be noted that m should be much less than M.

(3) Take samples n times from N training samples using put-back-sampling to form a training set. This process is called the bootstrap method. In addition, the unselected samples are used for prediction and the error is evaluated.

(4) For each node, m features are randomly selected, and the decision of each node in the decision tree is determined based on these features. According to these m characteristics, the optimal splitting mode is calculated.

(5) Each tree will grow completely without pruning and may be adopted after building a normal tree classifier.

(6) Repeat the above steps to form a random forest; the result is determined by the voting of trees in the random forest.

### 2.3. Data Acquisition

**Figure 3:**
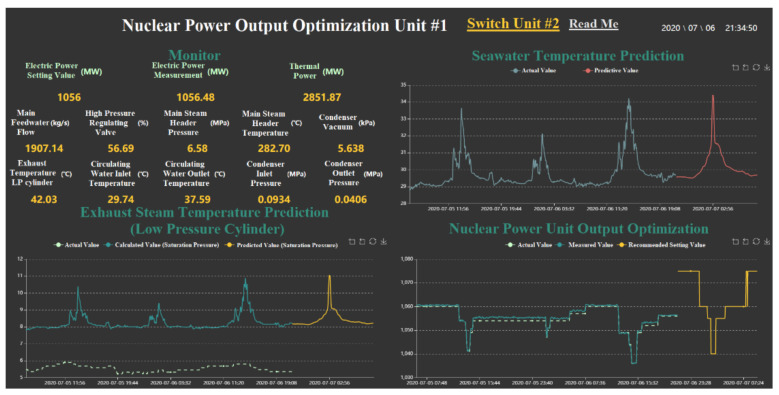
Monitoring and Forecast Program of Nuclear Power Unit.

In this paper, data are collected from the real-time database of unit 1 and unit 2 of the Qinshan nuclear power plant in Zhejiang Province, China. The steam turbine of this nuclear power plant is a saturated-steam single-shaft three-cylinder four-exhaust-intermediate-reheater half-speed nuclear power turbine manufactured by Dongfang Steam Turbine Manufacturer. The data are one sampling point per minute with each sampling containing 200 different types of parameters. The screenshot of the nuclear power monitoring program is shown in Figure 3.

The research object of this method is the sensor of the thermal system, with no consideration of the fault of the nuclear power unit itself. Therefore, the premise of this method is that the sampled data should be from the non-fault nuclear power units, so as to avoid the interference of parameter fluctuation caused by the fault of the nuclear power unit itself on the fault diagnosis results of the model. The method includes the following steps:

(1) In normal operation, the N sensors to be diagnosed are resampled K times, the sampling interval is Δt, and the sample length is L. The normal operation data sample $Q_{1}$ is obtained. The data label named “stable operation” is added to each sample in $Q_{1}$.

(2) In peak load operation, the sampling mode of 1) is repeated to obtain the peak load operation data sample $Q_{2}$. The data label named “peak load operation” is added to each sample in $Q_{2}$.

(3) On the basis of the actual operation data and the sensor fault simulation model proposed in reference [20], the fault signal simulation of the typical faults of four kinds of sensors is completed. Repeat the sampling mode of 1), obtain the data samples for each fault, and add the corresponding fault data label for each data sample. The sample set of fault 1 is named $Q_{31}$, the data label is “Catastrophic Failure (or Complete Failure)”; the sample set of fault 2 is named $Q_{32}$, the data label is “Precision Degradation Failure”; the sample set of fault 3 is named $Q_{33}$, the data label is “Drift Failure”; the sample set of fault 4 is named $Q_{34}$, the data label is “Constant Deviation Failure”.

(4) The Improved-ICA algorithm is applied to $Q_{total}=\left[Q_{1},Q_{2},Q_{31},Q_{32},Q_{33},Q_{34}\right]$. Obtain the physical source signal $S_{total}=\left[S_{Q1},S_{Q2},S_{Q31},S_{Q32},S_{Q33},S_{Q34}\right]$ and noise signal $E_{total}=\left[E_{Q1},E_{Q2},E_{Q31},E_{Q32},E_{Q33},E_{Q34}\right]$ under these five working conditions.

(5) Take the physical source signal $S_{total}$ and noise signal $E_{total}$ as the input value of the random forest model, then use the bootstrap method to select the training subset.

(6) According to the minimum mean square deviation, divide the left and right subtrees of the regression tree, carry out recursive calculation until the termination conditions are met, and obtain the random forest regression model by training the random forest model.

(7) Substitute the test subset into the random forest regression model to obtain the final prediction results for each sample and compare the actual value to evaluate the accuracy of the model.

## 3. Fault Diagnosis Process

According to the improved ICA algorithm and the random forest algorithm mentioned above, the fault diagnosis model is established. First of all, the signal obtained by the sensor is decomposed by the improved ICA and the characteristic sub-signal is extracted. Second, the decomposed characteristic sub-signal and noise signals are converted into training samples. Next, the labeled training samples are substituted into the random forest algorithm to complete the training, and the fault diagnosis model is obtained. Finally, the accuracy of the model is verified.

The steps of the model are as follows:

Signal analysis and feature extraction (completed by improved ICA)

1. Input original signal;
2. Completes the blind source separation by improved ICA;
3. Obtain the separated signal feature and remove the noise;
4. Taking the signal feature as one of the input values of the classifier;

Fault diagnosis (completed by random forest)

5. Take the signal feature and fault labels as the total samples;
6. The total samples are divided into training samples and test samples;
7. Initialize the random forest model;
8. Input training samples and complete the training;
9. Pass verification? If not, adjust the RF parameters and return to step 8;
10. Output the trained model;
11. Select the test sample as the input value of the trained model;
12. Input test samples and complete the test;
13. The test results are output and compared.

**Figure 4:**
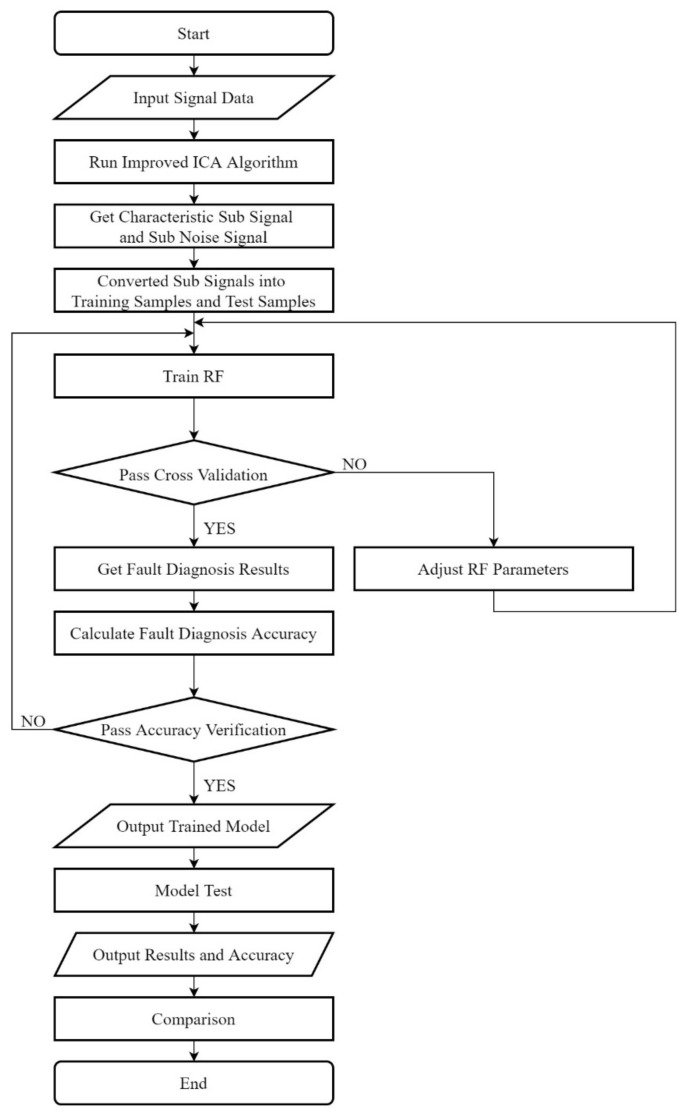
Fault Diagnosis Flow Chart.

The flow chart of the fault diagnosis model is shown in Figure 4.

### 3.1. The Result of Improved-ICA Algorithm

In order to verify the effectiveness of the fault diagnosis method, the data in the SIS real-time database of a 1211 MW nuclear power plant are analyzed. During the peak load operation of the nuclear power plant, the parameter signal of reheated steam pressure is abnormal, and the deviation between the collected sample data and the actual experience value is large; thus, the reheat steam pressure sensor is taken as the sensor to be diagnosed.

Apart from the reheat steam pressure, the fluctuation of the main-steam flow, low-pressure exhaust pressure, condenser temperature and other parameters also exceed the specified threshold. However, the fluctuation of these parameters is not as apparent as that of reheat steam pressure, especially the research on the fluctuation of main-steam flow also involving the finite element calculation of venturi pipe flow field. Another group of our research team is currently studying the fluctuation of the main steam flow. This study is based on the finite element calculation of the venturi flow field, which is also related to the vibration and cracking of the tube. Owing to its complexity, this is not analyzed in this manuscript. Considering the length of the paper and the research direction, these parameters are not discussed in this paper. Our team will focus on this part of the study in the next manuscript.

**Figure 5:**
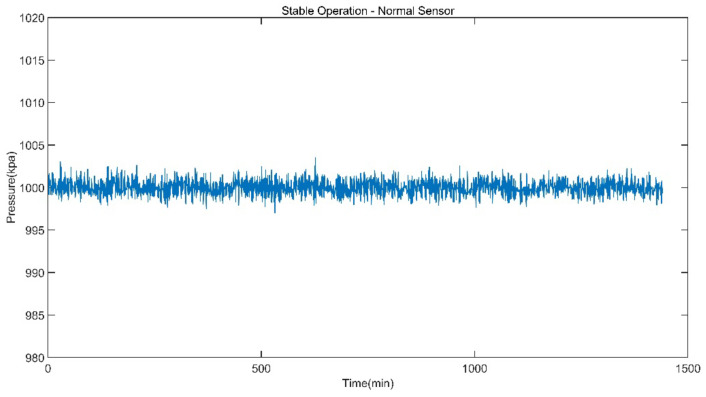
Reheat Steam Pressure of Stable Operation.

**Figure 6:**
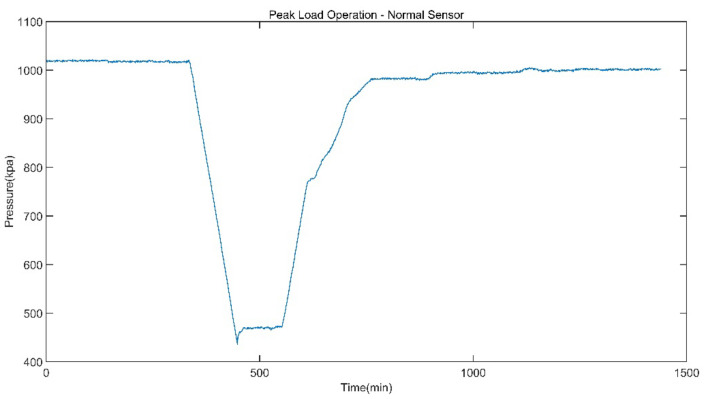
Reheat Steam Pressure of Peak Load Operation.

The data of reheat steam pressure under stable operation and peak load operation are shown in Figure 5 and Figure 6.

The improved ICA model is used for the blind source separation of the signal and outputs the separation result signals. In the figure, a total of four window graphs are listed from top to bottom, including the first separated principal component signal, the second separated principal component signal, the third separated principal component signal and the noise signal.

**Figure 7:**
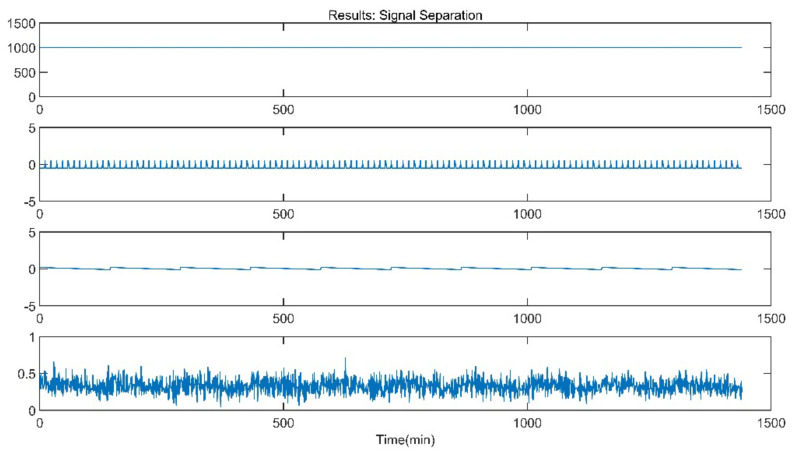
Separation Result (Stable Operation).

**Figure 8:**
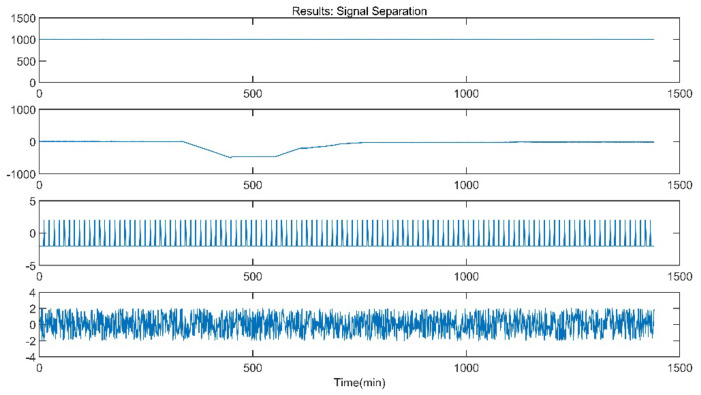
Separation Result (Peak Load Operation).

The separation results when the sensor works normally and the unit maintains stable operation are presented in Figure 7. When the sensor works normally and the unit is in peak load regulation, the separation result is shown in Figure 8.

When the sensor works normally, the first independent principal component extracted by the improved ICA is relatively stable, which is an approximately linear signal with a little burr noise. In stable operation, the second and third principal component signals are almost weak pulse signals, and the amplitude of the noise signal is small. In peak load operation, the second main component signal changes from the pulse signal to the section signal, and there are large negative value changes consistent with the time when the unit load fluctuation occurs. The amplitude of the third principal component signal and the noise signal increases, and the pulse form of the third principal component signal is more obvious. It can be seen that the pulse signal with the second weight degenerates into the third weight signal when the unit is in peak load regulation, while the parameter fluctuation signal caused by the load change in the unit evolves into the second weight signal.

In the thermal system sensors, the classical sensor faults can be divided into four categories: Catastrophic Failure, Precision Degradation Failure, Drift Failure and Constant Deviation Failure. There is no obvious Constant Deviation Failure in the sensor of this nuclear power station, thus this paper only studies the first three classical faults.

**Figure 9:**
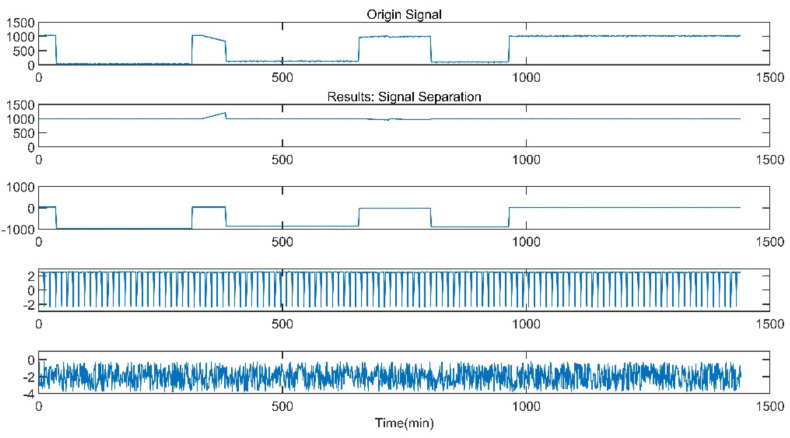
Separation Result of Catastrophic Failure.

**Figure 10:**
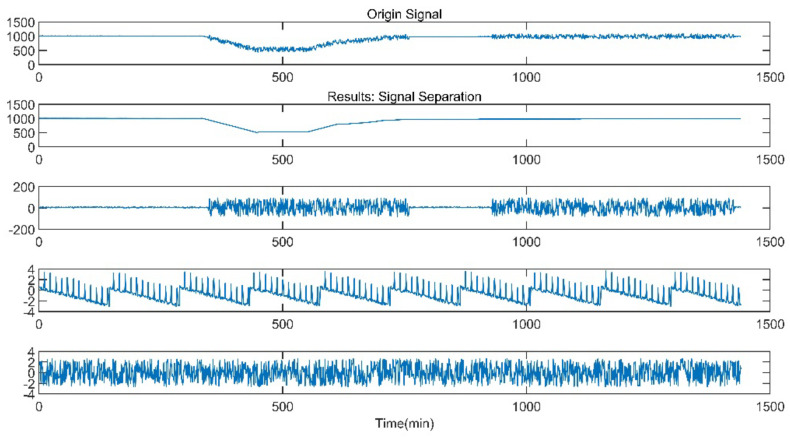
Separation Result of Precision Degradation Failure.

**Figure 11:**
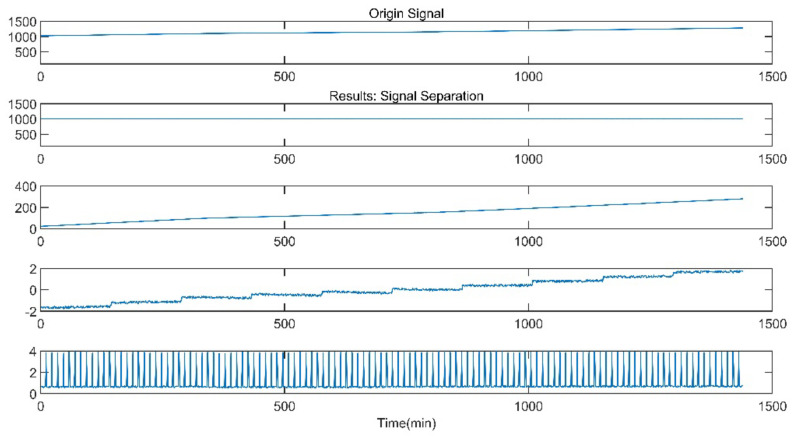
Separation Result of Drift Failure.

(1) When the sensor works in conditions of Catastrophic Failure, the measured value of the sensor remains unchanged for a certain period, and the measured value usually deviates from the actual experience value. During the peak load operation, the thermal parameters vary considerably according to the load fluctuation, and the measured values will not change in a certain period after the peak load regulation. The nuclear power unit was overhauled in March 2018 and August 2019. It was found that the sensor was in the state of Catastrophic Failure in both maintenances. After extracting this part of data, the sample data are analyzed. The signal separation result is shown in Figure 9. The negative value of the second principal component signal in Figure 9 corresponds to the Catastrophic Failure of the sensor. The Catastrophic Failure of the sensor will lead to the generation of a zero value signal. Due to the existence of a large number of zero value signals, the pulse form of the third principal component signal is more complex.

(2) When the accuracy of the sensor declines, the measured value of the sensor will fluctuate around the real value. Generally, the fluctuation range outstrips the maximum allowable value of the sensor error, and the fluctuation is irregular and periodic. During a peak load operation, the thermal parameters experience dramatic changes. From July 2019 to January 2020, the nuclear power unit experienced several incidents of excessive fluctuation. The field staff temporarily installed measuring points and the occurrence of Precision Degradation was verified. This paper selects a part of the fault sample data for analysis. The signal separation result is shown in Figure 10. Owing to the drop of precision, the third principal component signal is composed of a stepped signal and pulse signal with a great number of burr noises. The amplitude of the noise signal is also relatively large.

(3) When Drift Failure occurs, the measured value of the sensor changes in a divergent manner with time, and the change is fast with a wide scope. A thermal performance test was completed in September 2016. During the test, the reheat steam pressure changed greatly and increased with time. After stopping the thermal performance test, it was found that the sensor had Drift Failure. The signal separation result is shown in Figure 11. The second principal component signal is an approximately linear signal. The third principal component signal also presents certain linear characteristics with a small number of step pulses.

### 3.2. Model Training

The physical source samples and noise samples obtained in Section 3.1 are taken as the input values (characteristic parameters) of the random forest model. The sensor failure and fault type are taken as the output values (target parameters) of the random forest model. 1,440,000 groups of samples are randomly divided into a training set (accounting for 60% of the total number of samples) and a test set (constituting 40% of the total number of samples).

The most important parameters in the random forest regression model include the number of regression trees $n_{tree}$, the maximum characteristic number of regression trees $m_{try}$, the maximum depth of regression trees $m_{depth}$, the minimum number of samples divided into internal nodes $m_{split}$, and the minimum number of samples in leaf nodes $m_{leaf}$. If $n_{tree}$ is too small, the model will be is prone to underfitting; if $n_{tree}$ is too large, the model will be prone to overfitting. If the number of samples in a node is less than $m_{split}$, it will not continue to select the best features to partition. $m_{leaf}$ is related to the pruning of the regression tree. If the number of leaf nodes is less than $m_{leaf}$, it will be pruned together with its sibling nodes. Pruning helps to improve the generalization ability of the random forest. The parameters of $n_{tree}$, $m_{split}$ and $m_{leaf}$ are adjusted to obtain a more accurate random forest regression model [26,27].

Wu et al. [28] studied the relevant parameters of random forests. When the number of samples ranged from one million to ten million, the recommended parameter combination was $m_{split}\in \left[2,11\right]$ and $m_{leaf}\in \left[2,11\right]$. It is considered that if the number of data samples reaches the level of a million and the number of trees reaches 100, the accuracy will be improved slightly if the number of trees continues to increase. Moreover, the training time increases exponentially, which is not conducive to computer calculation.

Firstly, the parameters of $m_{split}$ and $m_{leaf}$ are optimized. The parameter range is given as $n_{tree}=100$, $m_{split}\in \left[2,11\right]$ and $m_{leaf}\in \left[2,11\right]$. Each model is trained by the training set. The evaluation index is out-of-bag error (oob error). Some of the training results are shown in Table 1.

Then, the parameter $n_{tree}$ is optimized. The parameter range is given as $n_{tree}\in \left[50,300\right]$ ($n_{tree}$ is a multiple of 10), $m_{split}=7$, $m_{leaf}=4$. Some of the training results are shown in Table 2.

The parameter combination used in this paper is: $m_{split}=7$, $m_{leaf}=4$, $n_{tree}=100$.

### 3.3. Experiments and Results

**Figure 12:**
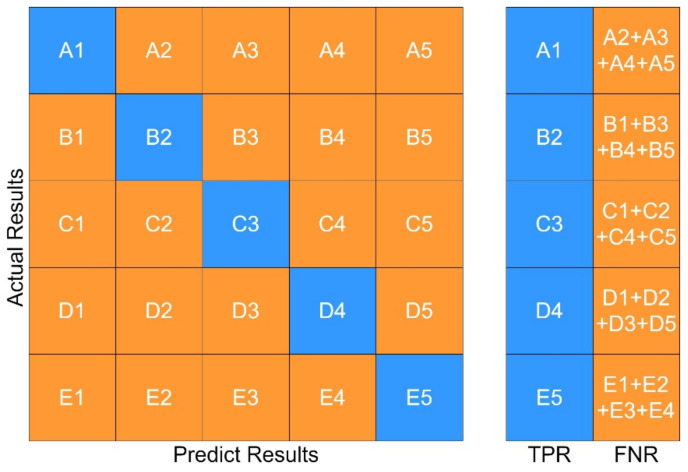
Confusion Matrix.

In the model training, four experiments are selected. Each model is trained by the training set and 20-fold cross-validation is used to evaluate the training effect of the model. The evaluation indexes are accuracy and calculation time. According to Krstajic et al. [29], a small number of K cannot meet the accuracy requirements, but at the same time, a larger number of K may not bring higher accuracy, although the overall trend is that the higher the number of folds is, the higher the accuracy is. To be sure, a large discount will cause a large computational overhead, causing a very long calculation time. Zhang et al. [30] suggested that when the total number of samples is very large, $K\approx log\;\left(n\right)$ and n/K>3d. n is the total number of samples and d is the number of features. It is proved by Jung et al. [31] that the stability of the 20-fold is better. It has quite good universality and can carry out better accuracy and calculation rate under different sample sizes. Based on the suggestions of these three references 20-fold cross-validation is used in the manuscript. The screenshot of the cross verification is shown in Figure 12. The training results of these four experiments are shown in Table 3.

(1) Experiment 1: Complete the fault diagnosis according to the methods and steps described in Section 3.1 and Section 3.2

(2) Experiment 2: The same training and test samples as those in Experiment 1 are used, but the random forest model is changed to KNN (K-Near-Neighbor) model;

(3) Experiment 3: Blind source separation in Section 3.1 is not performed. Using the observation signal as the input value, the fault diagnosis is completed according to the contents of Section 3.2;

(4) Experiment 4: The blind source separation in Section 3.1 is not performed. The observation signal is used as the input value, and the KNN model is used to complete the fault diagnosis.

**Figure 13:**
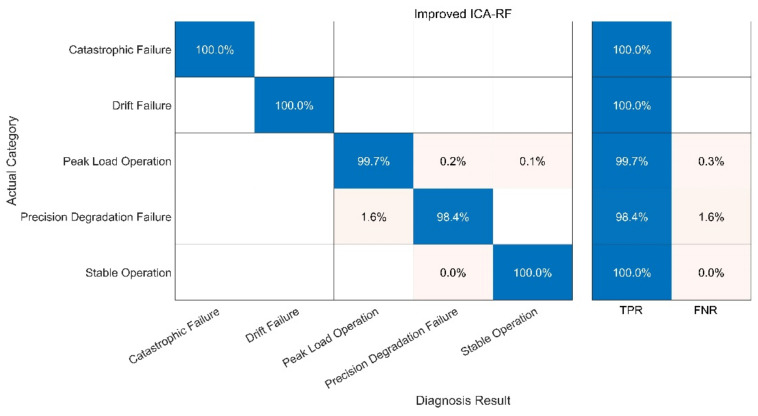
Fault Diagnosis Result of ICA-RF.

**Figure 14:**
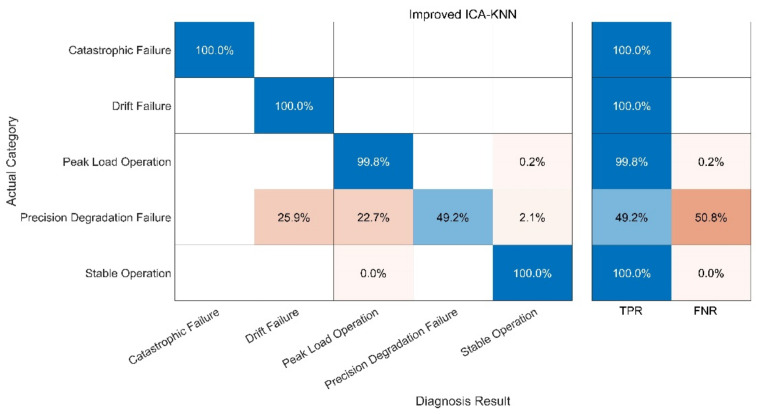
Fault Diagnosis Result of ICA-KNN.

**Figure 15:**
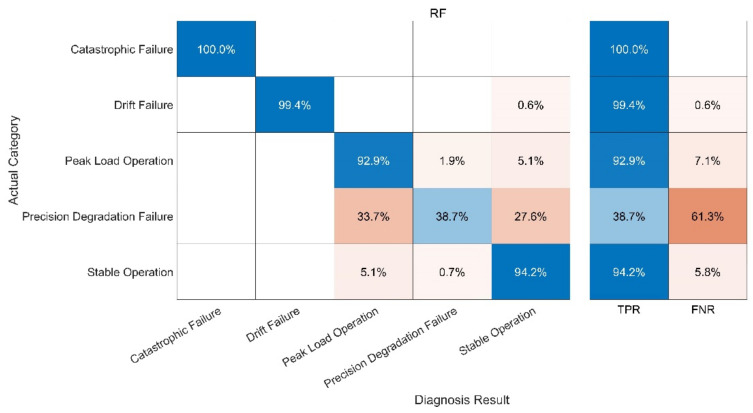
Fault Diagnosis Result of RF-Only.

**Figure 16:**
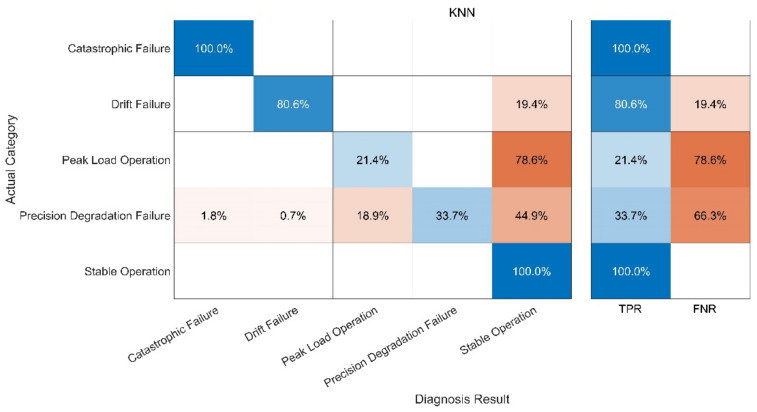
Fault Diagnosis Result of KNN-Only.

The fault diagnosis of the sample is carried out under the specified parameters. The following three groups of experiments have been carried out. In order to better evaluate the diagnosis results, a confusion matrix is used to compare the diagnosis results. The following definitions exist in the confusion matrix, and the details are given in Table 4 and Figure 13, Figure 14, Figure 15, Figure 16:

(1) If the positive class is predicted to be a positive class, it is a True Positive (TP).

(2) If the positive class is predicted to be a negative class, it is a False Negative (FN).

(3) If the negative class is predicted to be a positive class, it is a False Positive (FP).

(4) If the negative class is predicted to be a negative class, it is a True Negative (TN).

The test is divided into four parts:

(1) Test 1: Improved ICA-RF Model. The results are shown in Figure 13;

(2) Test 2: Improved ICA-KNN Model. The results are shown in Figure 14;

(3) Test 3: RF Model. The results are shown in Figure 15.

(4) Test 4: KNN Model. The results are shown in Figure 16.

The analysis and discussion of the test results will be carried out in detail in Section 4.

## 4. Discussion

Compared with the other three methods, the performance of the KNN-Only algorithm was the worst. The diagnostic accuracy of the KNN-Only algorithm for peak load operation was very low, and the classification effect of peak load operation and stable operation was not ideal. In addition, the KNN-Only algorithm was almost unable to identify the fault with reduced accuracy, and the diagnosis accuracy rate of the fault was only 33.7%. There were some deficiencies in the identification of data drift fault, and the accuracy rate was 80.6%. After blind source separation and feature extraction using improved ICA, the correct rate of the ICA-KNN algorithm for peak load operation increased to 99.8%, while the accuracy rate of data drift fault diagnosis was improved to 100%. The recognition ability of the fault with a reduced accuracy was promoted slightly, and the accuracy rate jumped to 49.2%, but the accuracy rate remained low.

Compared with the KNN algorithm, the RF algorithm showed a better performance. The recognition rate of the RF-Only algorithm outstripped that of the KNN-Only algorithm greatly, being close to that of the ICA-KNN algorithm. However, the RF-Only algorithm also had a poor identification accuracy standing at only 38.7%. Among all the methods, the ICA-RF algorithm showed the best performance. The diagnostic accuracy of the ICA-RF algorithm for complete failure and data drift fault was 100%, and the diagnostic accuracy of peak load operation was more than 99.7%. The accuracy of the model was 98.4% and it could not be diagnosed by the other three methods. Obviously, the improved ICA-RF algorithm model proposed in this paper could more quickly and accurately identify the working state and fault type of sensors in the peak shaving nuclear power thermal system.

In fact, when the observation signal is directly input into KNN, the characteristics of the peak load operation signal and stable operation signal are not significant at the initial stage of the peak load operation. In addition, some fault signals and peak load operation signals are similar to each other. These two factors cause serious interference in the KNN which uses spatial Euclidean distance to complete sample classification. When the signal features are vague, KNN cannot correctly identify the signal features, leading to low accuracy of diagnosis. RF can separate small-scale fluctuations on account of its use of the decision tree algorithm. However, the parameters of the peak-load-regulation nuclear power units fluctuate greatly, and the mixed-mode of signals is complex, creating a high demand for the accuracy of the decision tree function, increasing the difficulty of the decision tree classification and greatly augmenting the training time required. After ICA, physical source signal and noise signal represent different local characteristics of the original signal. Therefore, when ICA decomposed data are trained, KNN and RF can better reflect the characteristics of signals and greatly improve the diagnostic accuracy of RF. With the help of the above analysis, the improved ICA-RF algorithm can grasp the characteristic information of the sensor signal correctly and effectively, so as to clearly identify the working state and fault type of the sensor.

**Figure 17:**
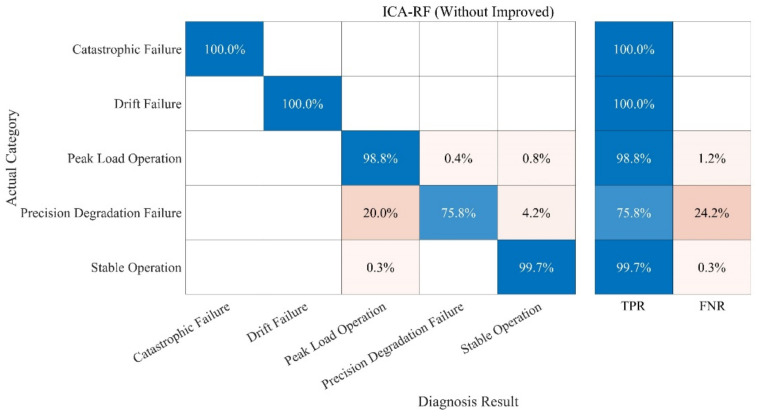
Fault Diagnosis Result of ICA-RF (Without Improved).

**Figure 18:**
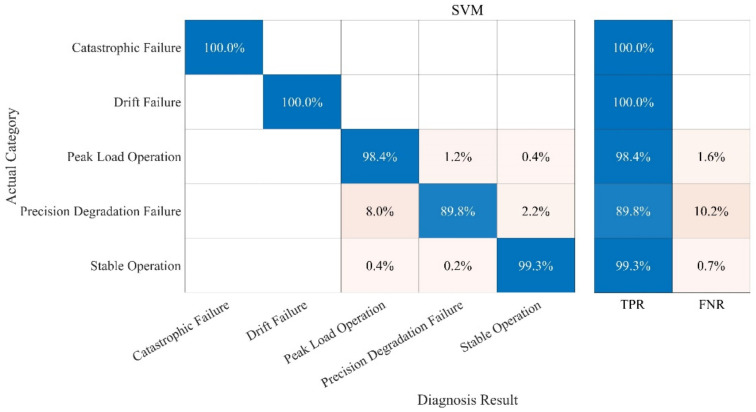
Fault Diagnosis Result of SVM.

In addition to the experiments of the above algorithm, in this paper, we also conducted experiments on the Without-Improved ICA-RF model and the Support Vector Machine (SVM) model, as shown in Figure 17 and Figure 18, respectively. The parameters of the SVM model are as follows: The kernel function is Gaussian kernel function; the nuclear scale is 3.2.

Compared with the fault diagnosis model proposed in this paper, the accuracies of the Without-Improved ICA-RF model and the SVM model are relatively low. More importantly, in terms of diagnosis time, the Without-Improved ICA model achieved 302 s, the SVM model achieved 542 s, and the Improved ICA-RF model achieved 44 s. The Improved ICA-RF model has more merits in terms of the accuracy and time taken for diagnosis.

**Figure 19:**
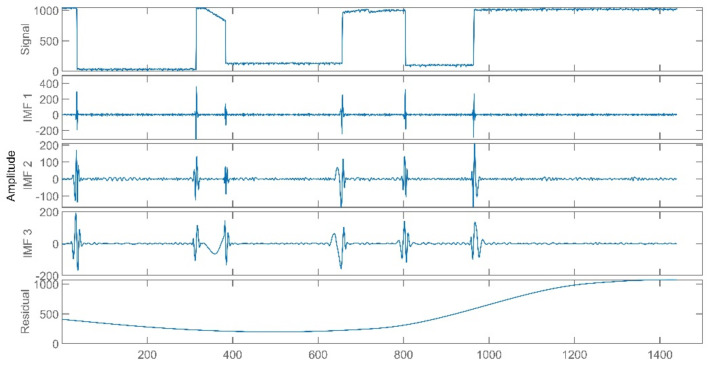
Catastrophic Failure Signal of EMD.

**Figure 20:**
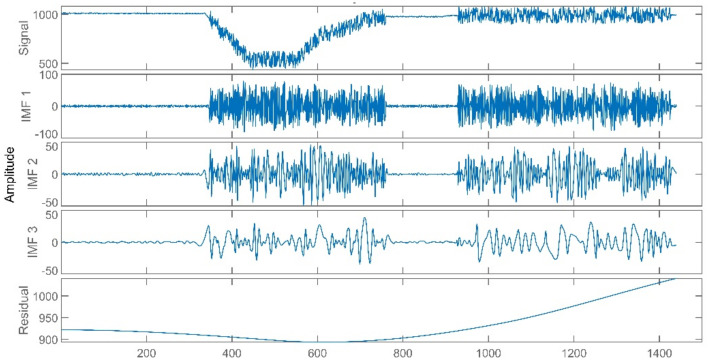
Precision Degradation Failure Signal of EMD.

**Figure 21:**
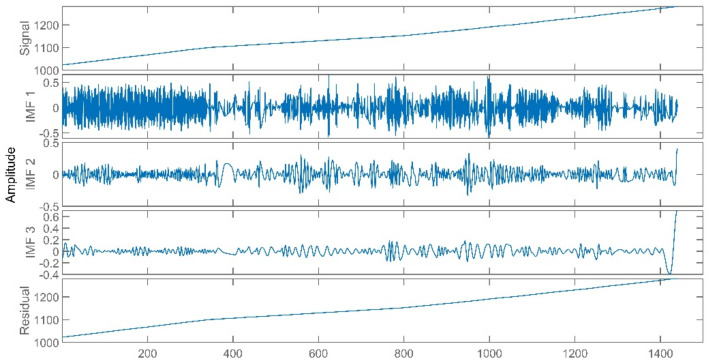
Drift Failure Signal of EMD.

**Figure 22:**
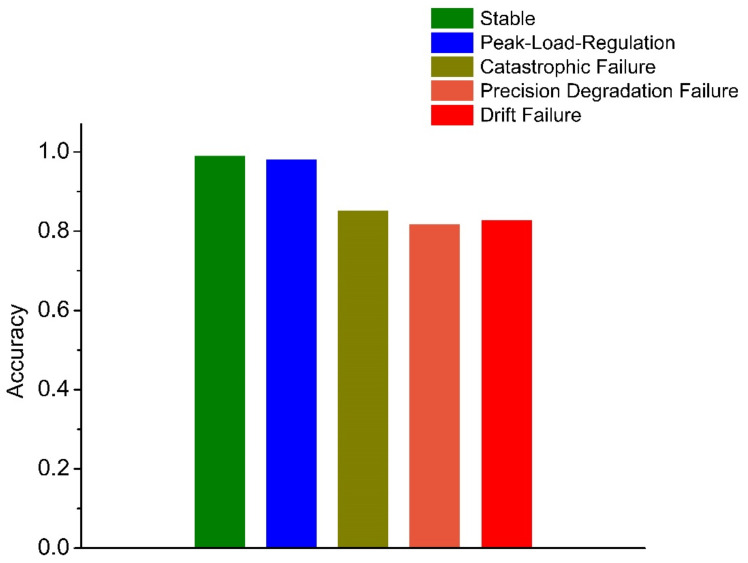
Result of EMD-RF.

**Figure 23:**
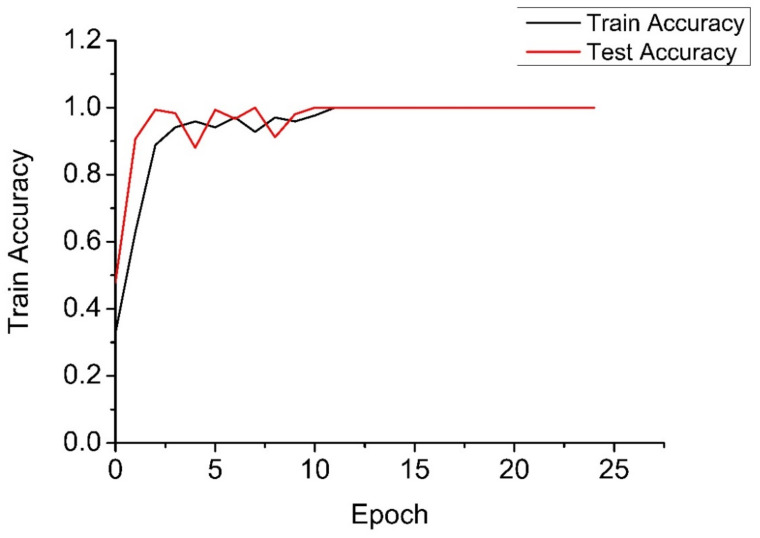
Result of CNN.

Through deeper consideration, we realized that the EMD is a signal decomposition method widely used in signal analysis, and the CNN is a very mature deep learning algorithm. We proposed some simple experiments of the EMD-RF [16] and the CNN [32,33]. The CNN is named Multi-Dimensional Signal Processing Convolution Neural Network (MDSP-CNN). In this paper, the MDSP-CNN runs on the basis of Python 3.8.8 and Tensorflow 2.3.0, and the editor is Jupyter 6.3.0. The signals processed by the EMD are shown in Figure 19, Figure 20, Figure 21. The result of the EMD-RF is shown in Figure 22. The result of the CNN is shown in Figure 23.

The overall comparison of the models is shown in Table 5. For different algorithms, the advantages of the improved ICA-RF are mainly reflected in accuracy and time-consuming. After the maximum approximate information, negative entropy was used to improve the ICA, compared with ICA-RF (Without Improved), the time-consuming of the algorithm proposed in this paper is significantly reduced from 302 s to 44 s; the ICA algorithm improves the classification accuracy. Compared with the RF and the KNN, the ICA-RF and the ICA-KNN improve the accuracy from 85% to 99.6% and from 67% to 89.78% respectively. Compared with some mature algorithms, such as the SVM and the EMD, the ICA-RF also has certain advantages. Compared with the SVM, the ICA-RF has just a small advantage in terms of accuracy, but the time consumption is nearly 500 s less than SVM; When the classifiers are both the RF, the total diagnostic accuracy of the EMD-RF is about 93%, but the diagnostic results of these three faults are poor, and the average accuracy is about 85%. In the diagnosis of those three faults, the model proposed in this paper has higher accuracy than the EMD-RF.

The diagnostic accuracy of the CNN is related to the epoch and the depth of network layers. A sufficiently large number of network layers can obtain very high accuracy. When the network depth is insufficient, the diagnosis accuracy of the CNN is low. However, the value of network depth needs research and experiment and is affected by sample size and data type. Although excessive network depth can reach a high accuracy, the calculation speed will be low, the code complexity will increase significantly, and the demand for computer hardware will be high. Though the ICA-RF has no advantage in accuracy compared with the CNN in certain epochs, the CNN needs a lot of experiments to determine the depth of network layers and the number of epochs, while the ICA-RF does not.

**Figure 24:**
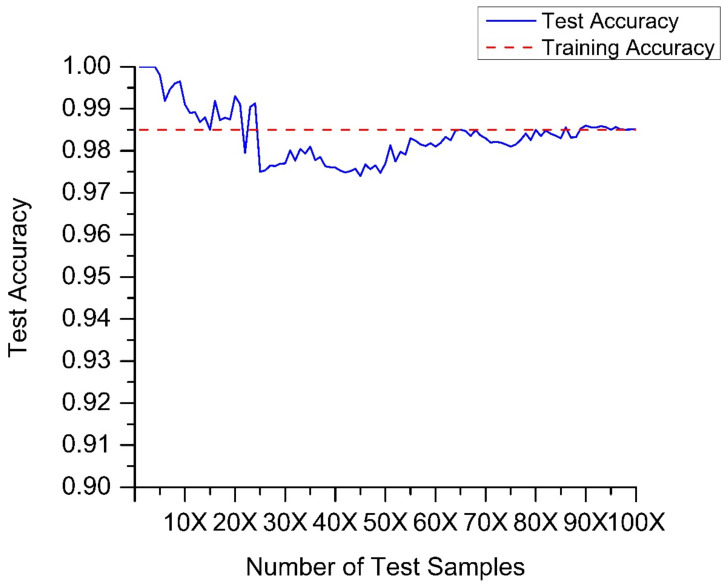
The Stability of Improved ICA-RF.

Finally, we studied and analyzed the stability of the improved ICA-RF algorithm. We expanded the test samples from 1 to 100 times (the number of 1-time samples = 1440) and calculated the accuracy of test samples of different sizes, as shown in Figure 24. The algorithm proposed in this paper reaches a high test accuracy when the number of samples is small, which is related to the small number of test samples. When the sample increases, the test accuracy decreases for a short period, but finally converges to the training accuracy. The model proposed in this paper has good stability and can be used for a large number of test samples.

The improved ICA-RF algorithm proposed in this paper has advantages in accuracy and time-consuming and can be applied to thermal sensor fault diagnosis of peak-load-regulation nuclear power units. The stability of this method is also good, and overfitting is not easy to occur when the sample size is large. Compared with the CNN, this method is simpler, does not need to optimize parameters through a large number of experiments, and is simpler and more convenient to use.

The shortcomings of the ICA mainly appear in the impact on the original data samples. Because the ICA decomposes the original signal, the total number of data will become larger. A large number of samples may increase the difficulty and time-consuming of calculation. The ICA algorithm itself is used to extract signal features, but the extracted features may not contain all the features of the original signal, so there is a case of changing the features of the original signal. The distortion of signal characteristics may affect the accuracy of the model. In addition, the ICA is inferior in algorithm complexity and computing time compared with some widely used signal analysis algorithms, such as median filtering algorithm and local mean decomposition algorithm. Moreover, the ICA is mainly used for signal separation and feature extraction of the non-Gaussian signals. When the noise is specific Gaussian noise, the effect is poor. The median filter is a nonlinear signal processing technology that can effectively suppress noise. The local mean algorithm has more advantages in analyzing the time-frequency distribution of signals. We will analyze these two methods in the next manuscript.

## 5. Conclusions

This paper proposed the Improved ICA algorithm, which can separate peak shaving signal from fault signal and obtain better signal characteristics. Additionally, according to the strong self-organization and self-learning ability of the random forest algorithm, a sensor fault diagnosis model based on the Improved ICA-RF algorithm is proposed. The experiment was carried out using MATLAB 2020b and the control group was set up. The results show that this method can distinguish peak shaving operation and sensor fault better and can identify the working state and fault type of sensor faster and more accurately than the method used in the control group.

The sensor fault diagnosis mentioned in this paper has been realized and is currently in test operation in the fourth power plant of the China National Nuclear Corporation (CNNC). Due to the security level of the CNNC and the strict management due to COVID-19, more data will be obtained and made public only after the acceptance of the project. In a follow-up study, we will expand the research case and research scope, and use data with a larger time span for our research, including the research results for sensor failure and steam turbine failure. This will be included in our next manuscript.

## Figures and Tables

**Table 1:** Parameter Optimization ($m_{split}$ and $m_{leaf}$).

$m_{split}$	$m_{leaf}$	Oob Error
7	2	17.69%
7	3	8.84%
7	4	3.21%
7	5	5.44%
5	4	9.17%
6	4	7.81%
8	4	6.93%
9	4	8.22%

**Table 2:** Parameter Optimization ($n_{tree}$).

$n_{tree}$	Accuracy	Time (s)
50	88.58%	38
100	97.04%	44
150	97.11%	89
200	94.77%	118
250	90.19%	142
300	85.16%	191

**Table 3:** The Results of Model Training.

Experiments		Training Accuracy	Training Time (s)
Exp. 1	ICA-RF	98.5%	70.606
Exp. 2	ICA-KNN	88.1%	36.199
Exp. 3	RF	77.2%	80.135
Exp. 4	KNN	68.4%	48.625

**Table 4:** Confusion Matrix.

		Predict		
		1	0	Total
Actual	1	True Positive **(TP)**	False Negative **(FN)**	Actual Positive **(TP+FN)**
	0	False Positive **(FP)**	True Negative **(TN)**	Actual Negative **(FP+TN)**
Total		Predicted Positive **(TP+FP)**	Predicted Negative **(FN+TN)**	**(TP+FN+FP+TN)**

True Positive Rate (TPR): TP/(TP+FN). False Negative Rate (FNR): FN/(TP+FN).

**Table 5:** Compare of Results.

Model	Test Accuracy	Test Time (s)
Improved ICA-RF	99.60%	44
Improved ICA-KNN	89.78%	29
RF	85.04%	59
KNN	67.14%	30
ICA-RF (Without Improved)	94.86%	302
SVM	97.50%	542
EMD-RF	92.87%	57
CNN (Epoch 1)	47.83%	22
CNN (Epoch 5)	98.33%	98
CNN (Epoch 10)	91.16%	173
CNN (Epoch 15)	99.98%	298

## Data Availability

Not applicable.

## Appendix A

**Pseudo Code: Improved ICA**

Start Step 1: Input data samples X Step 2: Calculate the number of rows Xrow and columns Xcol Define matrix size function as size () $\left[Xrow,\;Xcol\right]=\mathrm{size}\left(X\right)$ Step 3: Calculate the average aveX Define average function as mean() $\mathrm{ave}X=\mathrm{mean}\left(X\right)$ Step 4: Remove mean values Define all 1 matrix function as ones() For i = 1 : Xrow X(i,:)= X(i,:)- aveX(i)*ones(1, Xcol); End For Step 5: Calculate the covariance Cx Define the covariance function as cov() $Cx=\mathrm{cov}\left(X,1\right)$ Step 6: Calculate the feature vector eigvector and the feature value eigvalue Define the feature vector function as eig() $\left[eigvector,eigvalue\right]=\mathrm{eig}\left(Cx\right)$ Step 7: Calculate the whiten matrix Z $Z=\;\frac{eigvector}{\sqrt{eigvalue}}*X$ Step 8: Iterative initialization Set iteration steps Maxcount Set convergence condition Critical Step 9: Initialize B and $G_{i}$ Define random function as rand() $B=\mathrm{rand}\left(Xrow\right)$ Set $G_{i}$ as nonlinear function Step 10: Iterative operation Define expectation function as E() $$B^{*}=E\left\{ZG_{i}\left(B^{T}Z\right)\right\}-E\left\{G_{i}\left(B^{T}Z\right)\right\}B$$ $$B=\frac{B^{*}}{{||B||}^{*}}$$ Define absolute value function as abs() Set count=0 If $\mathrm{abs}\left(B-B^{*}\right)\;\&\;\mathrm{abs}\left(B+B^{*}\right)>Critical$ Then count=count+1 If count= Maxcount Then print(“Unconvergent”) Break Else Goto Step 9 End If Else Output B Break End If End
